# Preoperative Very-Low-Calorie Ketogenic Diet Versus Low-Calorie Diet in Bariatric Surgery: A Prospective Comparative Study

**DOI:** 10.3390/nu18101484

**Published:** 2026-05-07

**Authors:** Farnaz Rahimi, Stefano Boschetti, Isabella Comazzi, Costanza Pira, Vanessa Giordano, Agnese Gambetta, Sonia Tarallo, Virginia Alberini, Alessio Naccarati, Mirko Parasiliti-Caprino, Valentina Ponzo, Rosalba Rosato, Simone Arolfo, Mario Morino, Simona Bo

**Affiliations:** 1 Clinical Nutrition Unit, Città della Salute e della Scienza Hospital, 10126 Torino, Italy; 2 Department of Medical Sciences, University of Torino, 10126 Torino, Italy; 3 Genetic and Molecular Epidemiology Unit, Italian Institute for Genomic Medicine (IIGM), IRCCS Candiolo, 10060 Torino, Italy; 4 Department of Clinical and Biological Sciences, University of Torino, 10100 Torino, Italy; 5 Department of Psychology, University of Torino, 10124 Torino, Italy; 6 Department of Surgical Sciences, University of Torino, 10126 Torino, Italy

**Keywords:** obesity, bariatric surgery, very-low-calorie ketogenic diet (VLCKD), low-calorie diet (LCD)

## Abstract

Background: The very-low-calorie ketogenic diet (VLCKD) is increasingly used before bariatric surgery (BS), but its effects on surgical and long-term outcomes remain unclear. Objective: The aim of this study was to compare the impact of a 4-week VLCKD with a 4-week low-calorie diet (LCD) on preoperative, perioperative and postoperative outcomes for up 12 months in patients undergoing BS. Methods: In this prospective study, 72 (*n* = 36: VLCKD; *n* = 36: LCD) patients (mean age 43.2 ± 10.6 years; BMI 45.6 ± 6.4 kg/m^2^; 87.5% female) submitted to sleeve gastrectomy were enrolled at a tertiary care center from 2022 to 2024. Results: No adverse events were detected with both diets. The VLCKD was associated with a greater preoperative median weight loss percentage (−5.5 vs. −2.6 kg, *p* < 0.001), BMI reduction (−2.6 vs. −1.2 kg/m^2^, *p* < 0.001), shorter hospital stay (3.0 ± 0.2 vs. 3.4 ± 0.9 days, *p* = 0.017), higher day-1 postoperative hemoglobin (12.7 ± 1.3 vs. 12.0 ± 1.2 g/dL, *p* = 0.024), and lower day-1 postoperative median *C*-reactive protein levels (9.7 vs. 13.4 mg/L, *p* = 0.042). These associations were confirmed in a multiple regression model, after adjustments for BMI at enrolment, age and sex. After 6 months, the VLCKD was associated with greater reductions in mean weight loss percentage (−24.9 ± 7.8 vs. −19.6 ± 9.4 kg, *p* = 0.012), BMI reduction (−11.7 ± 4.4 vs. −8.7 ± 3.9 kg/m^2^, *p* = 0.003), neck circumference (−4.9 vs. −3.6 cm, *p* = 0.027) and lower aminotransferase levels. At 12 months, VLCKD patients maintained significant advantages over the same variables, except for neck circumference. Conclusions: A short preoperative VLCKD was safe and was associated with greater short-term weight reduction compared with the LCD, with potential benefits extending to early postoperative recovery and 1-year outcomes.

## 1. Introduction

The very-low-calorie ketogenic diet (VLCKD)—recently renamed very-low-energy ketogenic therapy (VLEKT)—is defined as a dietary regimen providing <800 kcal/day, with carbohydrate intake <50 g/day and moderate protein content, inducing nutritional ketosis [1,2,3]. It is primarily used for rapid weight loss in patients with obesity, including those with comorbidities, such as joint and cardiometabolic diseases, because of the substantial short-term reductions in body mass index (BMI), waist circumference, glycated hemoglobin (HbA1c), insulin resistance, blood pressure, and triglyceride values, with a favorable safety profile when medically supervised [3,4]. Weight loss during very-low-calorie ketogenic diets is driven by both caloric restriction and the metabolic effects of carbohydrate deprivation [2,3,4]. In the early phase, reduced carbohydrate intake leads to glycogen depletion, which is associated with a rapid loss of intracellular water and contributes to initial weight reduction [2,3]. As ketosis develops, ketone bodies become alternative energy substrates, promoting lipolysis and increased fat oxidation, thereby contributing to fat mass loss [3,4,5,6,7]. Moreover, the transition toward efficient ketone utilization (“keto-adaptation”) may vary among individuals, depending on factors such as metabolic flexibility, insulin sensitivity, and baseline nutritional status, highlighting the inter-individual variability in response to ketogenic interventions [8]. VLCKD protocols are used also before bariatric surgery, i.e., the most effective therapy for inducing long-term weight loss [9,10]. Over 2–4 weeks of a VLCKD, patients typically achieve 5–10% loss in body weight and up to a 20–30% reduction in liver volume, with improvements in glycemic and lipid profiles and no significant adverse effects [5,6,7]. A recent metanalysis of 17 studies found the highest preoperative weight loss (−8.62 kg) and BMI reduction (−5.75 kg/m^2^) with a VLCKD compared to low-calorie diets (LCDs) and very-low-calorie diets (VLCDs) [11]. Furthermore, prospective studies and clinical trials have demonstrated that short-term VLCKD interventions (typically 2–8 weeks) significantly reduced hepatic steatosis, as measured by ultrasonography, vibration-controlled transient elastography or magnetic resonance imaging, as well as improved liver function tests and metabolic parameters [5,6,7,12,13,14,15,16,17,18]. Visceral adipose tissue accumulation and enlarged liver have been reported to obstruct the surgical field and increase the risk of complications; thus, the preoperative loss of abdominal fat may confer technical advantages during surgery [19]. However, the benefits of pre-surgery weight loss are still a controversial topic, with few data suggesting a favorable impact in mitigating complications, but an uncertain level of evidence [9,20,21,22,23,24,25,26,27,28,29]. Furthermore, in addition to the undeniable benefits, bariatric surgery also carries early risks such as bleeding, infection, anastomotic leak, venous thromboembolism, pulmonary complications, prolonged hospital stay, and, less commonly, perioperative mortality [9,11]. To date, only one study has evaluated the effects of a VLCKD on short-term postoperative outcomes [30]; therefore, no conclusions can be drawn on its impact on early post-surgery complications [11]. On the other hand, other regimens, such as VLCDs and LCDs, showed a reduced postoperative hospital stay and lower surgery time, even if with a low level of evidence [11,28,31]. Finally, all available studies assessed the impact of different dietary regimens on pre-bariatric outcomes, while post-bariatric follow-up was either not considered or limited to a few days post-surgery [5,6,7,12,13,15,30].

The present prospective study aimed to compare, in patients with severe obesity, the effects of two 4-week preoperative dietary regimens—a standard LCD and a VLCKD—on surgical and metabolic outcomes, with assessments extending up to 1 year after bariatric surgery.

## 2. Materials and Methods

### 2.1. Participants

Patients were enrolled at the Clinical Nutrition Unit of the “Città della Salute e della Scienza” Hospital of Torino (Northern Italy). All patients were recruited between January 2022 and December 2024 and were managed by the same multidisciplinary bariatric team. Inclusion criteria were age > 18 and ≤65 years, BMI > 35 kg/m^2^ with at least one comorbidity (such as type 2 diabetes mellitus (T2DM), arterial hypertension, and sleep apnea) or BMI ≥ 40 kg/m^2^, many unsuccessful attempts to lose weight, and eligibility for bariatric surgery, according to international guidelines [32]. Exclusion criteria were: age > 65 years, secondary causes of obesity (e.g., hypothalamic diseases or other endocrine diseases), inability to participate in prolonged medical follow-up, risk of eating disorders, as assessed by a psychiatrist, presence of associated conditions impacting on weight loss (e.g., bariatric surgery in prevision of transplantation), kidney failure (creatinine levels > 1.8 mg/dL), mild to severe liver failure, type 1 diabetes mellitus, prolonged QT interval (>0.44 ms), cardiac arrhythmias, chronic use of diuretics, persistent diarrhea, recent history of heart attack, transient ischemic attack or stroke within the last 12 months, pregnancy and breastfeeding, malignancies, psychiatric disorders, allergies to milk proteins, and inability to give informed written consent.

### 2.2. Design of the Study

This was a prospective, non-randomized, comparative clinical study conducted in routine bariatric practice.

### 2.3. Ethical Issues

The study was approved by the local Ethics Committee (protocol number 0085704, date 14 September 2020) and was in accordance with the Declaration of Helsinki’s principles. Patients gave their informed consent to participate in the study.

### 2.4. Dietary Intervention

According to the usual clinical practice of our hospital, participants were visited by a multidisciplinary team of healthcare professionals experienced in the management of patients undergoing bariatric surgery, including a surgeon, an anesthetist, a psychiatrist, a physician specialized in clinical nutrition, and a dietitian. The presence of eating behaviors, previous attempts at weight loss, and the presence of comorbidities were ascertained before surgery. All patients received verbal and written dietary, exercise and behavioral recommendations about a healthy lifestyle, plus a written personalized dietary plan [33]. Patients who met the inclusion criteria were asked by two trained dietitians to choose whether to follow the standard dietary plan provided by the Clinical Nutrition Unit (an LCD) or the VLCKD.

In accordance with routine clinical practice, eligible patients received standardized nutritional counseling and balanced information regarding both dietary options. Based on informed personal preference, patients selected a preoperative dietary regimen: the standard low-calorie diet (LCD) provided by the Clinical Nutrition Unit or the very-low-calorie ketogenic diet (VLCKD). No randomization was performed.

The LCD was an individually prescribed diet in line with the Mediterranean diet composition (45–55% carbohydrates, <10% sugars, 30% fats, <10% saturated fats, 15–25% proteins, and 20–30 g fiber). Total energy intake was individually prescribed based on estimated energy requirements to achieve a daily deficit of 500–1000 kcal relative to estimated total energy expenditure [33]. Basal metabolic rate (BMR) was estimated using the Harris–Benedict predictive equation, a widely used method for estimating energy requirements in clinical practice [34]. Total energy expenditure (TEE) was calculated by applying a physical activity level (PAL) coefficient of 1.4, based on the low physical activity reported by participants. Suprabasal energy expenditure was not directly measured but indirectly accounted for through the applied PAL coefficient.

The VLCKD (Supplementary Table S1) was a commercial dietary plan (Named^®^ s.r.l., Lesmo, Italy) with the following composition: 750 kcal, 40 g carbohydrates (21.3%), 30 g lipids (36.0%), and 80 g proteins (42.7%). The protocol consisted of structured meal replacements distributed across three main meals and two daily snacks, including protein-based products such as soups, omelets, pasta, or bars, combined with low-carbohydrate vegetables (150 g/day) and a limited amount of extra-virgin olive oil (20 g/day). Beverages included water or sugar-free drinks (1.5–2 L/day), while coffee or tea without added sugar was allowed. Patients were encouraged to take trace elements and to drink at least 2 L/day of water.

Both diets must be strictly adhered to for 4 consecutive weeks, during which no dietary changes are permitted. Adherence was assessed through self-reported dietary records which were reviewed during structured weekly telephone consultations with trained dietitians and further verified during in-person visits. Compliance was considered satisfactory when participants reported adherence to the prescribed caloric intake and macronutrient distribution throughout the 4-week period. In the VLCKD group, adherence was additionally monitored by recording the consumption of prescribed meal replacement products.

In both groups, 150 min of moderate physical activity per week was recommended, including approximately 20 min of brisk walking per day (11 to 13 on the Borg Rating of Perceived Exertion scale) [35]. Furthermore, quitting smoking before surgery was mandatory.

Patients from both groups were assessed by a physician specialized in clinical nutrition and two trained dietitians before surgery, after the 4-week dietary regimens.

A minimum interval of 48 h was required between the end of the VLCKD and surgery, when carbohydrates were reintroduced and patients were transitioned to the LCD for a few days. On the first postoperative day, a blood sample was drawn to measure hemoglobin, white blood cells, and high-sensitivity *C*-reactive protein (hs-CRP). After surgery, all participants followed a standardized post-bariatric nutritional protocol and received dietary counseling during follow-up visits, during which dietary habits and adherence to recommendations were regularly assessed. Patients from both groups had to follow a 7-meal liquid diet for 4 weeks, and then a 5-meal soft/smooth diet for the following month. From the third month post-surgery onwards, foods with normal consistency were allowed. The prescribed energy amount was based on the Harris–Benedict formula, considering a caloric restriction of 500–1000 kcal, with 50% carbohydrates, 28% lipids, and 22% proteins.

Patients were followed up at our unit at 1, 3, 6, 12 and 24 months after surgery. Daily protein (whey proteins) and vitamin supplementation were prescribed starting from hospital discharge for the first 2 months (whey proteins) and 12 months (vitamins) after surgery; thereafter, supplementation was individualized according to specific needs.

During each visit, patients were weighed, and dietary habits were assessed. During the 6- and 12-month visits, a blood sample was drawn to evaluate metabolic and nutritional parameters.

### 2.5. Sleeve Gastrectomy (SG)

The surgical technique was standardized and never changed during the study period. Pneumoperitoneum was established through a Veress needle placed in the Palmer’s point and SG was performed using five trocars. The dissection and the isolation of the greater curvature from the gastro-colic and gastro-splenic ligaments were carried out using a radiofrequency sealing device (LigaSure, Medtronic, Dublin, Ireland). The gastric sleeve was calibrated on a 36 Fr oro-gastric bougie and the staple line extended 5 cm from the pylorus up to the angle of His, in accordance with the Fifth International Consensus Summit [36]. An adequate compression of gastric tissue was obtained before stapling in order to reduce edema, ensure precise wall overlap, and position metallic points correctly, as supported by Bellanger et al. [37]. The linear staplers applied were Covidien Endo GIA Tri-staple™ (Medtronic, Dublin, Ireland) and Ethicon Echelon Flex™ Endopath Stapler (Ethicon Endo-Surgery Inc., Cincinnati, OH, USA). The choice of the cartridges depended on the tissue thickness and the surgeon’s experience. An intraoperative leak test with methylene blue was routinely performed.

### 2.6. Postoperative Course

On the first postoperative day, patients underwent routine blood tests, including a complete blood count and measurement of hs-CRP levels, and started a liquid diet. If no complications occurred, discharge was planned on the 3rd postoperative day. Postoperative monitoring at the intensive care unit (ICU) was planned for patients with severe obstructive sleep apnea (OSA) or non-modifiable risk factors, such as severe heart disease. Postoperative monitoring was also required in case patients experiencing an intraoperative complication, including significant bleeding or respiratory issues.

Then, patients were followed up by surgeons at 6, 12, 24, 36, 48 and 60 months from surgery, reporting the percentage of excess weight loss, the onset of symptoms of gastroesophageal reflux disease or dysphagia and the remission of medical morbidities, defined as no need for pharmacological therapy.

### 2.7. Measurements

Before surgery, all patients underwent to the following examinations:

- Assessment of dietary intake by the same trained dietitians.
- Collection of blood samples to centrally measure fasting blood levels of glucose, glycated hemoglobin, triglycerides, creatinine, aspartate aminotransferase (AST), alanine aminotransferase (ALT), γ-glutamyl transferase (GGT), folic acid, vitamin B12, iron, transferrin, ferritin, and hs-CRP.
- Anthropometric measurements, including weight, height, waist and neck circumference, body fat mass and fat-free mass by bioelectrical impedance analysis (BIA).
- Measurements of blood pressure.
- Performance of esophagogastroscopy and liver ultrasound.

A timeline of the study assessments is provided in Supplementary Figure S1.

Blood sample analyses (fasting glucose, triglycerides, creatinine, AST, ALT, GGT, and hs-CRP) were repeated 6 and 12 months after surgery, while liver ultrasound was performed only at 12 months.

Weight and height were measured with patients wearing light clothes and no shoes by a digital scale with a capacity of 300 kg (Wunder Sa.Bi.srl, Monza, Italy) and the Stadiometer SECA 220 measuring rod (Hamburg, Germany), respectively. Waist and neck circumferences were evaluated by a non-stretchable flexible anthropometric measuring tape midway between the lowest rib and the iliac crest and under the cricoid cartilage, respectively. Fat mass (FM) and fat-free mass (FFM) were determined by single- frequency bioelectrical impedance (BIA 101, Akern, Florence, Italy). All measurements were performed using the same device and estimates were derived according to the manufacturer’s equations.

Laboratory measurements were centralized and performed according to standard methods.

Diagnoses of T2DM and arterial hypertension were made by general practitioners or the physicians of the multidisciplinary team in accordance with guidelines. OSA was hypothesized in the presence of suggestive symptoms and an intermediate to high-risk score on the STOP-Bang questionnaire [38]. The diagnosis was subsequently confirmed by a sleep-expert neurologist using additional exams, such as in-laboratory polysomnography or unattended home sleep apnea testing, as necessary. A trained radiologist diagnosed liver steatosis via ultrasound examination. Anemia was defined by the hemoglobin thresholds established by the World Health Organization: hemoglobin < 13 g/dL for males and <12 g/dL for females [39].

Percentage of total weight loss (TWL%) was calculated as follows:

(Preoperative weight − postoperative weight)/preoperative weight × 100

Percentage excess weight loss (%EWL) was calculated as follows:

(Preoperative weight − Postoperative weight)/(Preoperative weight − Ideal weight) × 100

with ideal weight defined as the weight corresponding to a BMI of 25 kg/m^2^.

Changes in variables (Deltas) were the difference between preoperative and postoperative values.

### 2.8. Statistical Analysis

Based on the difference in hospital stay reported by Albanese et al. [30], and assuming a between-group difference of 1 day with a standard deviation of approximately 1 day, 21 patients per group would provide 90% power (two-sided α = 0.05). To account for potential dropouts, the planned sample size was increased to 40 participants per group. Variables are presented as mean ± standard deviation (SD) or, for non-normally distributed variables, as the median (25th; 75th quartile). The Kolmogorov–Smirnov test was used to assess normality. Between-group comparisons were performed using the Chi-square test, Student’s *t*-test, or Mann–Whitney U test, as appropriate. The association between the VLCKD and postoperative outcomes was assessed by multiple regression analysis, adjusted for age, sex, and preoperative BMI.

Longitudinal associations between dietary groups (VLCKD vs. LCD) and changes in anthropometric, hemodynamic, and biochemical outcomes were assessed using linear mixed-effects models with a random intercept for each participant to account for repeated measurements. Fixed effects included time (baseline, 6 months [T6], and 12 months [T12]), the Diet × Time interaction, and covariates (baseline BMI, age, sex, and percentage of pre-bariatric weight loss). Models were estimated by maximum likelihood, and results are reported as unstandardized coefficients (B) with corresponding *p*-values. Separate models were fitted for each outcome (BMI, body weight, neck circumference, systolic and diastolic blood pressure, hs-CRP, glucose, triglycerides, AST, ALT, GGT, and creatinine).

For each model, the overall effects of time and the Diet × Time interaction were first evaluated (global tests). Post hoc contrasts at individual follow-up time points were interpreted only when appropriate. The inclusion of random intercepts was supported by likelihood ratio tests versus standard linear models, confirming subject-level heterogeneity.

Univariate comparisons at individual time points were performed for descriptive purposes only. Linear mixed-effects models were considered the primary inferential approach for repeated-measures data; therefore, no formal correction for multiple testing was applied to descriptive comparisons, which should be interpreted as exploratory.

Additional sensitivity analyses are reported in the Supplementary Materials. These included (i) models incorporating 1-month follow-up data and (ii) intention-to-treat analyses with imputation of the small proportion of missing values. These analyses yielded results consistent with the primary findings, indicating no material change in the overall conclusions.

All statistical tests were two-sided, and a *p*-value < 0.05 was considered statistically significant. Analyses were performed using Stata 18.0 (StataCorp LLC, College Station, TX, USA).

## 3. Results

### 3.1. Participants

The first 80 patients (40 on VLCKD and 40 on LCD) who gave their informed consent to participate and met the inclusion criteria were enrolled in the study. At enrolment, age was 43.9 ± 10.7 years and BMI was 46.3 ± 7.2 kg/m^2^, with 87.5% of participants being females (Supplementary Table S2). The clinical and anthropometric characteristics of the two groups were similar and not significantly different, as well as pre-enrolment macronutrient composition (15.9% and 15.6% proteins, 30.4% and 30.9% lipids, and 52.7% and 52.3% carbohydrates, respectively, in the VLCKD and LCD groups).

Subjects who underwent gastric bypass surgery, four for each group, were excluded due to the different metabolic effects of this procedure compared with SG. Therefore, the data from the 36 patients in each group who underwent SG were analyzed.

The baseline characteristics of these individuals were not significantly different (Table 1).

### 3.2. Preoperative Outcomes

All participants completed the 4-week preoperative dietary intervention (completion rate: 100%), with no dropouts, diet switching, or discontinuations due to intolerance or adverse events.

Patients from the VLCKD group had significantly higher weight loss, as well as larger reductions in BMI, waist and neck circumference, and percentage of fat mass compared with the LCD group (Table 2).

### 3.3. Perioperative Outcomes

Mean operative time was slightly shorter, though not significantly different, in the VLCKD group. The duration of hospital stays, post-surgery hs-CRP, and percentages of anemia were significantly lower, while 1-day post-surgery hemoglobin levels were higher in the latter group (Table 2). A few mild complications occurred after surgery, and no adverse cardiopulmonary events were reported in both groups. A transfer to the ICU was necessary to administer high-flow oxygen in two cases and to monitor high-risk patients for 24 h in the remaining cases. After discharge, two patients were visited at the emergency room for wound problems without requiring hospitalization. No further complications occurred in the two groups during the 12-month follow-up period. In a multiple regression model, the VLCKD was significantly associated with 1-day postoperative hemoglobin, hs-CRP levels and the number of hospital days, after adjustments for age, sex, and preoperative BMI (Table 3).

### 3.4. Long-Term Postoperative Outcomes

At 6 months postoperatively, all patients attended a follow-up visit, although only 83.3% (60/72) agreed to blood sample collection. At this time point, patients in the VLCKD group showed greater reductions in body weight, BMI, neck circumference, and transaminase levels than those in the LCD group, whereas metabolic variables, creatinine, and hs-CRP did not differ significantly between groups, although values were numerically more favorable in the VLCKD group (Table 4; Supplementary Figures S2–S5).

At 12 months after bariatric surgery, the VLCKD group continued to show a greater percentage of weight loss, a larger reduction in BMI, and lower transaminase levels compared with the LCD group (Table 5; Supplementary Figures S2–S5).

Longitudinal analyses were performed using linear mixed-effects models including time, the Diet × Time interaction, and relevant covariates (Table 6). Overall, significant time effects were observed for BMI, body weight, neck circumference, systolic and diastolic blood pressure, triglycerides, GGT, and hs-CRP, indicating a substantial improvement over time across the entire cohort. In contrast, the Diet × Time interaction was significant only for selected outcomes, suggesting an additional longitudinal benefit of a preoperative VLCKD primarily on anthropometric measures and selected biochemical parameters.

BMI decreased by 8.7 units at 6 months and 12.6 units at 12 months (both *p* < 0.001). Patients receiving the preoperative VLCKD experienced an additional reduction of 3.0 units at 6 months (*p* = 0.001) and 3.4 units at 12 months (*p* < 0.001), independently of baseline BMI, age, sex, and percentage pre-bariatric weight loss. Similarly, body weight decreased by 22.9 kg and 33.3 kg at 6 and 12 months, respectively (both *p* < 0.001), with a further reduction in the VLCKD group of 8.1 kg at 6 months (*p* = 0.001) and 9.1 kg at 12 months (*p* = 0.001). Neck circumference also declined over time (−3.6 cm at 6 months and −3.8 cm at 12 months, both *p* < 0.001), with a significantly greater decrease in the VLCKD group (−1.3 cm at 6 months, *p* = 0.024; −1.6 cm at 12 months, *p* = 0.011).

Systolic blood pressure decreased by 7.6 mmHg at 6 months and 14.0 mmHg at 12 months (*p* = 0.003 and *p* < 0.001, respectively), while diastolic blood pressure decreased by 4.0 mmHg and 7.0 mmHg (*p* = 0.025 and *p* < 0.001, respectively), confirming a significant overall time effect without a significant Diet × Time interaction. Mean glucose levels did not show a significant overall time effect, although a greater reduction was observed in the VLCKD group at 6 months (−13.3 mg/dL; *p* = 0.014). Triglycerides decreased significantly over time (−26.7 mg/dL at 12 months; *p* = 0.003), without an additional dietary effect.

Among liver enzymes, AST showed a significant additional reduction in the VLCKD group at 6 months (−7.4 U/L; *p* = 0.023), whereas ALT did not show significant longitudinal changes. GGT decreased significantly over time (approximately −9 U/L at both 6 and 12 months; *p* ≤ 0.001), without a significant Diet × Time interaction. hs-CRP also declined significantly in the overall cohort (−6.3 mg/L at 6 months and −7.5 mg/L at 12 months; both *p* < 0.001), with no additional effect of the VLCKD. Creatinine was lower at baseline in the VLCKD group (B = −0.109 mg/dL; *p* = 0.018), whereas neither time nor Diet × Time effects were significant in adjusted models.

Among the covariates, higher baseline BMI was positively associated with BMI, body weight, neck circumference, systolic blood pressure, and hs-CRP. Male sex was associated with greater body weight and larger neck circumference. Older age was positively associated with systolic blood pressure and inversely associated with hs-CRP. The percentage pre-bariatric weight loss was positively associated with BMI, body weight, and hs-CRP, and inversely associated with creatinine.

Sensitivity analyses, including models incorporating 1-month follow-up data and intention-to-treat analyses with imputation of missing values (Supplementary Table S3), produced results that were virtually identical to the primary analyses, with no changes in the direction, magnitude, or statistical significance of any findings.

## 4. Discussion

A short preoperative VLCKD was associated with greater weight loss and improvements in anthropometric parameters compared with an LCD, with potential benefits extending into the early postoperative course and partially persisting up to one year after surgery.

### 4.1. Preoperative Outcomes

Both the VLCKD and LCD groups were well-matched at baseline in terms of age, sex distribution, BMI, and anthropometric and biochemical characteristics, ensuring the comparability of the results. After 4 weeks of dietary intervention, patients on the VLCKD achieved greater reductions in body weight, BMI, and waist and neck circumference, as well as a higher percentage of excess weight loss compared to the LCD. The weight loss was mainly at the expense of fat mass. The interpretation of weight loss during the VLCKD should take into account that part of the initial reduction may be related to glycogen depletion and the associated loss of intracellular water, a well-recognized early effect of carbohydrate restriction [2,3,8]. However, in our study, the greater reduction in fat mass observed in the VLCKD group supports a true effect on adiposity beyond these early changes. Following carbohydrate restriction, hepatic ketogenesis is activated and ketones become alternative energy substrates. This metabolic shift typically occurs within a few days, although the timing of this transition (“keto-adaptation”) may vary among individuals depending on factors such as insulin sensitivity and metabolic flexibility [8]. In addition, the marked caloric restriction inherent to a VLCKD induces a substantial negative energy balance, which contributes to reductions in visceral adiposity and improvements in metabolic parameters, including insulin sensitivity. These effects are well documented in previous studies on ketogenic dietary interventions in obesity, which have demonstrated rapid weight loss and favorable anthropometric changes, particularly in pre-surgical settings [5,6,7,12,13,14,15,16,17,18,40,41]. Importantly, no adverse events were reported during the dietary intervention, confirming the safety of VLCKDs in the usual clinical practice, consistent with the existing literature supporting the use of this regimen in carefully monitored patients [4,5,6,7,12,13,15,30]. Additionally, our results confirm the feasibility and effectiveness of this dietary regimen in a real-world clinical setting, with the possibility of achieving significant benefits in the short term.

### 4.2. Perioperative Outcomes

Ketones have been reported to exert several metabolic effects, including serving as alternative energy substrates and potentially modulating inflammatory and oxidative pathways. Experimental studies suggest that ketosis may influence inflammatory signaling and cellular metabolism; however, these mechanisms were not directly investigated in the present study [42].

In our cohort, patients undergoing the VLCKD exhibited lower postoperative hs-CRP levels and better preservation of hemoglobin concentrations. While obesity is characterized by chronic low-grade inflammation, particularly within visceral adipose tissue [19], the mechanisms underlying the observed differences remain speculative.

Preclinical, translational and clinical studies have suggested that β-hydroxybutyrate may modulate inflammatory signaling pathways and oxidative stress responses, potentially contributing to improved metabolic profiles [8,42,43,44,45,46,47,48,49]. Additionally, ketogenic diets have been associated with improvements in insulin sensitivity and reductions in adiposity, which may indirectly influence systemic inflammatory markers [40,41]. Nevertheless, because molecular inflammatory pathways were not directly assessed in our study, these proposed mechanisms should be interpreted as hypothetical explanations rather than demonstrated effects. Further mechanistic investigations are warranted to clarify the biological pathways underlying the clinical observations.

The reduction in postoperative hs-CRP levels in our patients from the VLCKD group is relevant because elevated CRP values within the first 1–3 days postoperatively have been associated with a higher risk of complications, while low levels showed a high negative predictive value for ruling out major morbidity [50,51,52]. No study has previously assessed the association of a pre-bariatric VLCKD on post-surgery CRP values to the best of our knowledge. Consistently, our patients undergoing a VLCKD experienced fewer postoperative complications compared to the LCD group. The low number of complications did not allow for significant differences between the groups. However, the difference in the length of stay was significantly shorter after the VLCKD. It is well known that a longer hospital stay is associated with several adverse consequences for both patients and healthcare systems, including increased risk of complications, unplanned hospital re-admissions, and substantial resource utilization [53,54]. These results were confirmed by multiple regression analysis, after taking into account preoperative BMI. Similarly, Albanese et al. found a lower percentage of patients requiring a >3-day hospital stay in patients submitted to a VLCKD before SG [30].

Our patients from the VLCKD group showed significantly higher hemoglobin levels at the first postoperative follow-up than those from the LCD group, confirming the results of Albanese et al. who compared a 3-week VLCKD with 3-week VLCD and found higher postoperative hemoglobin levels in the former (13.1 ± 1.2 vs. 12.7 ± 1.5 g/dL, *p* = 0.04) [30]. This effect was correlated with a greater preoperative BMI reduction, which is associated with improved surgical outcomes, including reduced drainage output [30]. The implicated mechanisms may be multifactorial: greater preoperative weight loss and improved metabolic and inflammatory profiles may reduce intraoperative blood loss and perioperative complications, thereby preserving postoperative hemoglobin. The VLCKD has also been shown to improve metabolic parameters and reduce liver size, which can facilitate surgery and potentially reduce surgical trauma [5,6,7,12,13,14,15,16,17,18,30]. However, in the absence of estimated blood loss data, these findings should be interpreted cautiously.

Overall, our results suggest that improved preoperative metabolic and anthropometric profiles due to the VLCKD may contribute to enhanced recovery. The low incidence of complications and absence of severe adverse events in both groups confirm the safety of bariatric surgery when preceded by this dietary regimen.

### 4.3. Long-Term Postoperative Outcomes

Using mixed-effects models adjusted for BMI at enrolment, age, sex, and the percentage of pre-bariatric weight loss, outcomes improved across the whole cohort over time, with the VLCKD conferring additional, clinically meaningful benefits on top of these common trends. The VLCKD was associated with greater reductions in BMI, body weight, and neck circumference by 6 months, and these advantages were maintained, though attenuated, at 12 months. Glycemia showed an early VLCKD advantage at 6 months, and AST displayed a selective early reduction, whereas blood pressure, triglycerides, GGT, and CRP improved over time irrespective of diet, consistent with weight-loss-driven effects. Renal safety was supported by the absence of adverse creatinine trajectories. The long-term univariate analysis aligns with these multivariable findings. At 6 months, VLCKD patients retained an advantage in weight, BMI, neck circumference, and transaminase reductions compared with the LCD, while other biochemical markers, including metabolic variables and creatinine, did not differ between groups. By 12 months, between-group differences in anthropometry had narrowed, yet the percentage of weight loss, the reduction in BMI, and lower transaminase levels remained significantly different, reinforcing both the efficacy and safety of a preoperative VLCKD over the longer term, with no evidence of renal impairment.

Persistently lower transaminases suggest a favorable hepatic effect of the VLCKD pathway, highly relevant in obesity-related liver disease. Although ketone bodies may exert hepatoprotective actions by modulating inflammation, fibrosis, and oxidative stress [55], it is unlikely that the one-month ketogenic phase alone explains the one-year differences; more plausibly, the maintenance of greater weight loss and metabolic improvement underlies the sustained hepatic signal. Nevertheless, once all patients undergo bariatric surgery, a powerful determinant of weight and metabolic status, the incremental long-term impact of the preoperative diet may be modest.

These results reopen the debate on whether preoperative weight loss influences long-term bariatric outcomes. Prior studies suggest small and inconsistent associations between greater preoperative weight loss and total weight loss up to 12 months, and possibly up to 5 years, after surgery [56,57,58], with limited and conflicting evidence for enhanced long-term comorbidity resolution or weight maintenance. Accordingly, pre-surgery weight reduction is not currently a mandatory prerequisite for bariatric surgery [9,20,21,22,23,24,25,26,27,28,29]. To our knowledge, no previous work has assessed the long-term consequences of a preoperative VLCKD; our findings therefore provide novel insights for pre-surgical optimization and underscore the need for prospective, mechanistic, and comparative studies to determine how distinct dietary protocols might shape long-term metabolic health and organ function after bariatric surgery.

### 4.4. Strengths and Limitations

This study has several strengths. To our knowledge, it is among a limited number of studies evaluating long-term postoperative outcomes, including hs-CRP levels, in patients undergoing bariatric surgery following a preoperative VLCKD. Additionally, the comparison with an LCD, whose role in perioperative outcomes remains debated, provides clinically relevant insight into preoperative nutritional strategies.

However, several limitations must be considered when interpreting the findings. First, the non-randomized design represents a major limitation, as participants self-selected their dietary regimen. This may have introduced selection bias and residual confounding, as unmeasured factors such as motivation, socioeconomic status, or prior dietary preferences could have influenced both group allocation and outcomes. Therefore, causal inferences regarding the effects of the dietary interventions cannot be established.

The two dietary interventions differed not only in macronutrient composition but also in delivery. The VLCKD protocol involved standardized pre-packaged meal replacement products, whereas the LCD required self-selection and preparation of foods. This difference represents a potential source of bias, as it may have influenced adherence, dietary consistency, and energy intake. In addition, differences in cost burden and logistical support between the two approaches may have affected compliance and outcomes independently of dietary composition.

Ketone bodies were not directly measured, and therefore the presence and degree of nutritional ketosis cannot be confirmed. As a result, attributing the observed effects specifically to ketosis remains speculative. The findings should be interpreted as reflecting the overall dietary intervention rather than a confirmed ketogenic metabolic state.

Although the sample size was adequate for the primary perioperative outcome, it may have been insufficient to detect smaller differences in secondary outcomes or to explore heterogeneity in postoperative responses. In addition, the single-center design may limit the generalizability of the findings.

Adherence to the dietary interventions was primarily assessed through self-report and may therefore be subject to reporting bias. Although the greater weight loss observed in the VLCKD group may suggest higher adherence, this cannot be definitively established.

Body composition was assessed only preoperatively and not systematically during follow-up, preventing evaluation of long-term changes in fat-free mass and other body compartments. Furthermore, body composition was estimated using single-frequency bioelectrical impedance analysis, which is subject to variability depending on the device and prediction equations used. Energy expenditure was estimated rather than directly measured, which may have introduced error in the assessment of energy balance and contributed to variability in weight loss outcomes. Not all patients consented to blood sampling during follow-up, which may have limited the ability to detect subtle biochemical differences; analyses using an ITT approach, including all participants initially enrolled, yielded results consistent with the primary findings.

Finally, intraoperative estimated blood loss was not systematically recorded, which may have influenced postoperative hemoglobin levels and limited the interpretation of hematological outcomes.

Overall, these limitations suggest that the findings should be interpreted with caution and considered hypothesis-generating. Randomized controlled trials with standardized intervention delivery, objective adherence measures, and direct metabolic assessments are needed to confirm these findings and better define their clinical applicability.

## 5. Conclusions

In summary, a short-term VLCKD before bariatric surgery was associated with greater preoperative weight loss and more favorable perioperative outcomes compared with a standard LCD, with some effects persisting up to one year. However, given the non-randomized design, and the lack of a direct measurement of ketosis, these findings should be interpreted with caution, are hypothesis-generating, and do not support causal conclusions. Further randomized multicenter studies with standardized interventions and direct metabolic assessments are needed to confirm these findings and to define the patient populations most likely to benefit.

## Figures and Tables

**Table 1:** Clinical characteristics at enrolment.

	Total	VLCKD	LCD	*p*
Number	72	36	36	
Males/females, n	9/63	5/31	4/32	0.722
Age, years	43.2 ± 10.6	42.9 ± 10.8	43.4 ± 10.6	0.834
Active smoking, n (%)	12 (16.7)	7 (19.4)	5 (13.9)	0.527
T2DM, n (%)	12 (16.7)	7 (19.4)	5 (13.9)	0.527
Treatment for T2DM, n (%)	8 (11.1)	5 (13.9)	3 (8.3)	0.453
Arterial hypertension, n (%)	24 (33.3)	12 (33.3)	12 (33.3)	-
Treatment for hypertension (%)	16 (22.2)	8 (22.2)	8 (22.2)	-
Liver steatosis, n (%)	54 (75.0)	28 (77.8)	26 (72.2)	0.586
OSA, n (%)	15 (20.8)	9 (25.0)	6 (16.7)	0.402
Systolic blood pressure (mmHg)	131.9 ± 13.6	131.7 ± 11.8	132.1 ± 15.4	0.898
Diastolic blood pressure (mmHg)	82.0 ± 8.7	81.7 ± 9.2	82.4 ± 8.7	0.742
Weight, kg	120.7 ± 19.3	123.5 ± 21.6	118.0 ± 16.5	0.234
Height, cm	162.6 ± 7.4	162.8 ± 8.8	162.5 ± 5.7	0.850
BMI, kg/m^2^	45.6 ± 6.4	46.5 ± 6.6	44.8 ± 6.3	0.259
Bioelectrical Impedance				
Intracellular water (L)	21.4 ± 3.1	21.8 ± 3.5	21.1 ± 2.7	0.338
Extracellular water (L)	24.7 ± 5.4	25.0 ± 5.5	24.4 ± 5.4	0.628
Fat-free mass (kg)	62.8 ± 11.0	63.9 ± 11.7	61.8 ± 10.5	0.425
Fat mass (kg)	59.0 ± 14.4	59.3 ± 14.9	58.7 ± 14.1	0.855
Waist circumference, cm	124.6 ± 12.4	127.1 ± 11.9	122.2 ± 12.5	0.095
Neck circumference, cm	39.1 ± 3.9	39.9 ± 4.2	38.4 ± 3.6	0.111
Fasting glucose (mg/dL)	94.4 ± 24.7	98.6 ± 32.5	90.3 ± 11.8	0.156
Glycated hemoglobin, mmol/mol	38.4 ± 6.9	39.1 ± 7.9	37.6 ± 5.7	0.375
Triglycerides (mg/dL)	124.9 ± 67.6	125.3 ± 63.0	124.4 ± 72.7	0.955
Creatinine, mg/dL	0.77 ± 0.18	0.75 ± 0.13	0.78 ± 0.23	0.410
AST, U/L	22.4 ± 8.9	22.5 ± 9.1	22.3 ± 8.8	0.937
ALT, U/L	28.2 ± 15.0	27.8 ± 12.6	28.6 ± 17.2	0.821
GGT, U/L	33.3 ± 21.4	34.9 ± 22.1	31.7 ± 20.8	0.529
Hemoglobin, g/dL	13.5 ± 1.2	13.5 ± 1.2	13.5 ± 1.2	0.806
Iron, µg/dL	73.0 ± 30.2	72.1 ± 21.2	73.9 ± 37.4	0.808
Transferrin, mg/dL	298.8 ± 47.0	293.0 ± 44.4	304.6 ± 49.5	0.296
Ferritin, ng/mL	57.5 (23.0; 104.5)	59.5 (23.0; 103.5)	55.5 (24.5; 104.5)	0.693 *
Folic acid, ng/mL	5.1 ± 2.7	4.9 ± 2.1	5.4 ± 3.3	0.569
Vitamin B12, ng/L	393.8 ± 158.8	392.8 ± 157.5	394.8 ± 162.4	0.958
White blood cells, (cells/µL)	7513.8 ± 2306.9	7504.4 ± 2215.3	7523.1 ± 2426.5	0.973
hs-CRP, mg/L	8.4 (4.1; 13.9)	8.7 (4.4; 13.4)	8.0 (3.3; 14.1)	0.698 *

Mean ± SD; median (25th; 75th quartile); * *p*-value by Mann–Whitney U test. Aspartate aminotransferase (AST), alanine aminotransferase (ALT), g-glutamyl transferase (GGT), high-sensitivity *C*-reactive protein (hs-CRP), low-calorie diet (LCD), obstructive sleep apnea (OSA), type 2 diabetes mellitus (T2DM), very-low-calorie ketogenic diet (VLCKD).

**Table 2:** Early surgical outcomes in the two groups.

	VLCKD	LCD	*p*
Preoperative anthropometric changes			
Delta weight, kg	−7.0 (−9.5; −5.7)	−3.0 (−4.1; −1.4)	<0.001 *
Percent weight loss before surgery, kg	5.5 (4.2; 7.6)	2.6 (1.2; 3.9)	<0.001 *
Delta BMI, kg/m^2^	−2.6 (−3.5; −2.0)	−1.2 (−1.7; −0.5)	<0.001 *
Delta waist circumference, cm	−7.0 (−11.0; −3.5)	−5.0 (−6.5; −2.0)	0.036 *
Delta neck circumference, cm	−2.0 (−2.0; −1.0)	−1.0 (−1.8; 0.0)	<0.001 *
Bioelectrical Impedance **			
Delta fat-free mass (kg)	−3.0 (−4.6; +0.7)	−1.0 (−2.8; +0.7)	0.246
Delta fat mass (kg)	−5.6 (−9.3; −2.5)	−2.5 (−4.6; −0.1)	0.011
Operation time, minutes	59.8 ± 18.2	68.9 ± 33.8	0.159
Postoperative characteristics during hospital stay			
1-day postoperative hemoglobin, g/dL	12.7 ± 1.3	12.0 ± 1.2	0.024
Anemia ***, n (%)	8 (22.5)	17 (47.1)	0.026
1-day postoperative white blood cells, cells/µL	10,367.8 ± 3917.6	10,983.9 ± 2832.0	0.447
1-day postoperative hs-CRP, mg/L	9.7 (4.0; 17.0)	13.4 (10.6; 21.9)	0.042 *
Fever/infections, n (%)	1 (2.8)	0 (0.0)	0.314
Wound dehiscence, n (%)	1 (2.5)	1 (2.5)	-
ICU after surgery, n (%)	2 (5.6)	5 (13.9)	0.233
Duration of hospital stay, days	3.0 ± 0.2	3.4 ± 0.9	0.017
Hospital stay >3 days, n (%)	1 (2.8)	7 (19.4)	0.024
Post-discharge complications			
Access to the emergency room, n (%)	0.0	2 (5.6)	0.152

Mean ± SD; median (25th; 75th quartile); * *p*-value by Mann–Whitney U test. ** Data available in 35 participants from both groups; *** hemoglobin < 13 g/dL (♂) and <12 g/dL (♀). High-sensitivity *C*-reactive protein (hs-CRP), low-calorie diet (LCD), very-low-calorie ketogenic diet (VLCKD).

**Table 3:** Association between VLCKD and post-surgery outcomes in a multiple regression model.

	Beta	SE	*p*
1-day postoperative hemoglobin			
VLCKD	0.706	0.276	0.013
Preoperative BMI (kg/m^2^)	−0.046	0.022	0.040
Age (years)	−0.009	0.013	0.491
Male sex	0.990	0.429	0.024
1-day postoperative hs-CRP			
VLCKD	−10.086	3.501	0.005
Preoperative BMI (kg/m^2^)	1.028	0.281	<0.001
Age (years)	−0.058	0.167	0.730
Male sex	13.281	5.441	0.017
Days of hospital stay			
VLCKD	−0.441	0.132	0.001
Preoperative BMI (kg/m^2^)	0.044	0.011	<0.001
Age (years)	0.006	0.006	0.314
Male sex	0.273	0.205	0.187

Very-low-calorie ketogenic diet (VLCKD).

**Table 4:** Clinical characteristics at 6 months after bariatric surgery.

	VLCKD	LCD	*p*
Number	36	36	
Delta weight, kg	−31.0 ± 12.0	−22.9 ± 10.6	0.003
Percent weight loss, kg	24.9 ± 7.8	19.6 ± 9.4	0.012
Percent excess weight loss, kg	55.9 ± 18.3	47.5 ± 26.2	0.122
Delta BMI, kg/m^2^	−11.7 ± 4.4	−8.7 ± 3.9	0.003
Delta waist circumference, cm	−22.0 (−30.0; −17.0)	−19.0 (−25.0; −12.0)	0.135 *
Delta neck circumference, cm	−4.9 ± 2.4	−3.6 ± 2.4	0.027
Systolic blood pressure (mmHg)	121.3 ± 11.5	124.4 ± 12.7	0.268
Diastolic blood pressure (mmHg)	77.2 ± 7.7	78.3 ± 6.5	0.511
Treatment for hypertension, n (%)	7 (19.4)	6 (16.7)	0.759
Treatment for T2DM, n (%)	1 (2.8)	0 (0.0)	0.314
OSA, n (%)	4 (11.1)	4 (11.1)	-
Fasting glucose, mg/dL **	81.0 ± 12.6	87.9 ± 15.4	0.064
Triglycerides, mg/dL **	94.5 ± 21.4	108.5 ± 35.8	0.071
Creatinine, mg/dL **	0.73 ± 0.15	0.77 ± 0.18	0.366
AST, U/L **	19.0 ± 6.7	26.6 ± 18.6	0.039
ALT, U/L **	20.7 ± 13.6	31.3 ± 24.7	0.044
GGT, U/L **	20.7 ± 8.1	22.3 ± 13.6	0.582
hs-CRP, mg/L **	2.0 (1.1; 4.7)	2.1 (0.6; 6.0)	0.836 *

Mean ± SD; median (25th; 75th quartile); * *p*-value by Mann–Whitney U test. ** Values available for n = 30 patients on VLCKD and n = 30 patients on LCD. Aspartate aminotransferase (AST), alanine aminotransferase (ALT), g-glutamyl transferase (GGT), high-sensitivity *C*-reactive protein (hs-CRP), low-calorie diet (LCD), obstructive sleep apnea (OSA), type 2 diabetes mellitus (T2DM), very-low-calorie ketogenic diet (VLCKD).

**Table 5:** Clinical characteristics at 12 months after bariatric surgery.

	VLCKD	LCD	*p*
Number	30	30	
Delta weight, kg	−42.1 ± 14.7	−33.6 ± 9.3	0.009
Percent weight loss, kg	33.6 ± 8.0	28.9 ± 8.5	0.030
Percent excess weight loss, kg	74.5 ± 18.0	70.4 ± 28.3	0.509
Delta BMI, kg/m^2^	−16.0 ± 5.3	−12.7 ± 3.4	0.006
Delta waist circumference, cm	−32.0 (−40.0; −27.5)	−27.5 (−37.0; −18.0)	0.075 *
Delta neck circumference, cm	−5.2 ± 3.2	−3.9 ± 2.5	0.100
Systolic blood pressure (mmHg)	116.8 ± 10.1	117.8 ± 12.3	0.732
Diastolic blood pressure (mmHg)	74.8 ± 9.6	75.2 ± 5.6	0.870
Treatment for hypertension, n (%)	4 (13.3)	4 (13.3)	-
Treatment for T2DM, n (%)	1 (3.3)	0 (0.0)	0.313
OSA, n (%)	3 (10.0)	3 (10.0)	-
Liver steatosis, n (%)	4 (12.5)	7 (21.9)	0.320
Fasting glucose, mg/dL	79.6 ± 6.7	84.7 ± 13.3	0.066
Triglycerides, mg/dL	84.7 ± 25.7	95.9 ± 27.0	0.106
Creatinine, mg/dL	0.73 ± 0.11	0.79 ± 0.23	0.214
AST, U/L	18.1 ± 4.2	23.6 ± 13.2	0.033
ALT, U/L	17.1 ± 7.3	23.5 ± 13.6	0.027
GGT, U/L	18.9 ± 6.5	22.1 ± 15.9	0.317
hs-CRP, mg/L	1.4 (0.8; 2.1)	1.2 (0.5; 4.2)	0.848 *

Mean ± SD; median (25th; 75th quartile); * *p*-value by Mann-Whitney U test. Aspartate aminotransferase (AST), alanine aminotransferase (ALT), g-glutamyl transferase (GGT), high-sensitivity *C*-reactive protein (hs-CRP), low-calorie diet (LCD), obstructive sleep apnea (OSA), type 2 diabetes mellitus (T2DM), very-low-calorie ketogenic diet (VLCKD).

**Table 6:** Adjusted coefficients (B), 95% confidence intervals (CIs), and *p*-values from linear mixed-effects models estimated by maximum likelihood.

VLCKD vs. LCD		BMI		Weight		Neck Circumference		Systolic Blood Pressure		Diastolic Blood Pressure		Hs-CRP	
		B (95% CI)	*p*-Value	B (95% CI)	*p*-Value	B (95% CI)	*p*-Value	B (95% CI)	*p*-Value	B (95% CI)	*p*-Value	B (95% CI)	*p*-Value
Baseline difference		1.5 (−0.1; 3.1)	0.072	5.1 (−0.9; 11.2)	0.097	1.2 (−0.1; 2.6)	0.081	0.5 (−5.8; 6.7)	0.884	−0.5 (−4.5; 3.5)	0.820	0.7 (−2.5; 4.0)	0.658
Time effect	6 mo	−8.7 (−9.9; −7.4)	0.000	−22.9 (−26.3; −19.4)	0.000	−3.6 (−4.4; −2.8)	0.000	−7.6 (−12.7; −2.6)	0.003	−4.0 (−7.5; −0.5)	0.025	−6.3 (−9.1; −3.5)	0.000
	12 mo	−12.6 (−13.9; −11.2)	0.000	−33.3 (−37.0; −29.6)	0.000	−3.8 (−4.6; −2.9)	0.000	−14.0 (−19.2; −8.7)	0.000	−7.0 (−10.7; −3.3)	0.000	−7.5 (−10.3; −4.7)	0.000
VLCKD and time interaction	6 mo	−3.0 (−4.8; −1.2)	0.001	−8.1 (−13.0; −3.2)	0.001	−1.3 (−2.4; −0.2)	0.024	−2.8 (−9.9; 4.3)	0.445	−0.4 (−5.4; 4.6)	0.870	−1.5 (−5.5; 2.4)	0.453
	12 mo	−3.4 (−5.3; −1.5)	0.000	−9.1 (−14.3; −3.9)	0.001	−1.6 (−2.8; −0.4)	0.011	−1.3 (−8.8; 6.2)	0.726	0.1 (−5.1; 5.3)	0.971	−0.8 (−4.8; 3.1)	0.680
Covariates	Baseline BMI	0.8 (0.8; 0.9)	0.000	1.9 (1.6; 2.3)	0.000	0.2 (0.1; 0.3)	0.000	0.3 (0.0; 0.6)	0.044	0.1 (−0.1; 0.3)	0.401	0.3 (0.1; 0.4)	0.000
	Age	0.0 (−0.0; 0.1)	0.051	−0.0 (−0.2; 0.2)	0.735	0.0 (−0.0; 0.1)	0.575	0.2 (0.0; 0.4)	0.034	0.1 (−0.0; 0.2)	0.083	−0.1 (−0.2; −0.0)	0.039
	Male Sex	1.2 (−0.3; 2.8)	0.122	13.3 (6.7; 19.9)	0.000	5.8 (4.4; 7.3)	0.000	3.3 (−2.5; 9.2)	0.264	3.2 (−0.3; 6.7)	0.076	−0.8 (−3.9; 2.3)	0.617
	%PWL	0.4 (0.1; 0.6)	0.002	1.0 (0.0; 2.0)	0.044	0.1 (−0.2; 0.3)	0.570	0.4 (−0.5; 1.3)	0.351	0.1 (−0.4; 0.6)	0.653	0.6 (0.1; 1.1)	0.011
**VLCKD vs. LCD**		**Glucose**		**Triglycerides**		**AST**		**ALT**		**GGT**		**Creatinine**	
		**B (95% CI)**	***p*** **-Value**	**B (95% CI)**	***p*** **-Value**	**B (95% CI)**	***p*** **-Value**	**B (95% CI)**	***p*** **-Value**	**B (95% CI)**	***p*** **-Value**	**B (95% CI)**	***p*** **-Value**
Baseline difference		7.0 (−3.5; 17.6)	0.192	−12.2 (−37.6; 13.1)	0.344	0.4 (−5.3; 6.0)	0.899	−0.6 (−8.6; 7.5)	0.889	1.7 (−7.8; 11.1)	0.728	−0.1 (−0.2; −0.0)	0.018
Time effect	6 mo	−2.7 (−10.3; 4.8)	0.477	−14.1 (−31.9; 3.8)	0.123	4.1 (−0.4; 8.6)	0.071	2.7 (−4.4; 9.7)	0.459	−9.2 (−14.5; −4.0)	0.001	−0.0 (−0.1; 0.0)	0.134
	12 mo	−5.7 (−13.3; 1.8)	0.134	−26.7 (−44.5; −8.8)	0.003	1.2 (−3.3; 5.7)	0.608	−5.2 (−12.2; 1.9)	0.152	−9.4 (−14.7; −4.2)	0.000	−0.0 (−0.1; 0.0)	0.439
VLCKD and time interaction	6 mo	−13.3 (−24.0; −2.6)	0.014	−11.7 (−36.9; 13.6)	0.366	−7.4 (−13.8; −1.0)	0.023	−9.6 (−19.6; 0.4)	0.061	−2.9 (−10.4; 4.6)	0.448	0.0 (−0.0; 0.1)	0.438
	12 mo	−9.8 (−20.5; 0.9)	0.071	−8.8 (−34.1; 16.4)	0.494	−5.3 (−11.7; 1.1)	0.102	−5.4 (−15.4; 4.6)	0.294	−4.5 (−11.9; 3.0)	0.242	0.0 (−0.1; 0.1)	0.974
Covariates	Baseline BMI	0.6 (0.1; 1.2)	0.024	0.4 (−0.9; 1.8)	0.556	0.2 (−0.1; 0.5)	0.181	0.2 (−0.2; 0.6)	0.299	0.1 (−0.4; 0.7)	0.623	0.0 (0.0; 0.0)	0.001
	Age	0.1 (−0.3; 0.4)	0.757	−0.4 (−1.2; 0.4)	0.350	−0.1 (−0.2; 0.1)	0.482	−0.0 (−0.2; 0.2)	0.905	−0.1 (−0.5; 0.2)	0.407	0.0 (−0.0; 0.0)	0.051
	Male Sex	7.0 (−4.3; 18.2)	0.224	20.8 (−6.2; 47.9)	0.131	4.1 (−1.6; 9.8)	0.158	3.0 (−4.7; 10.6)	0.447	3.9 (−6.8; 14.6)	0.475	0.1 (−0.0; 0.2)	0.159
	%PWL	0.0 (−1.7; 1.7)	0.991	−3.5 (−7.6; 0.6)	0.091	0.2 (−0.7; 1.1)	0.640	0.2 (−0.9; 1.3)	0.733	−0.3 (−1.9; 1.3)	0.688	−0.0 (−0.0; −0.0)	0.037

Fixed effects included diet (VLCKD = 1, LCD = 0), time (baseline [reference], 6 months, 12 months), and the Diet × Time interaction, with adjustment for baseline BMI, age, sex, and percentage of pre-bariatric weight loss (%PWL). “Baseline difference” represents the VLCKD–LCD difference at baseline. “Time effect” indicates the change over time from baseline in the reference group (LCD). “VLCKD × Time interaction” represents the additional change in the VLCKD group beyond the time effect (i.e., difference-in-differences). Accordingly, the between-group difference at 6 and 12 months equals: baseline difference + VLCKD × Time interaction (6 or 12 months). The within-VLCKD change equals: time effect + VLCKD × Time interaction. Covariates are shown as adjusted associations with each outcome within the same models. Units: BMI (kg/m^2^); body weight (kg); neck circumference (cm); systolic and diastolic blood pressure (mmHg); hs-CRP (mg/L); glucose, triglycerides, and creatinine (mg/dL); AST, ALT, and GGT (U/L). Abbreviations: B, beta; 95% CI, 95% confidence intervals; VLCKD, very-low-calorie ketogenic diet; LCD, low-calorie diet; %PWL, percentage of pre-bariatric weight loss; hs-CRP, high-sensitivity *C*-reactive protein; mo, months.

## Data Availability

The data presented in this study are available on reasonable request from the corresponding author. The data are not publicly available due to privacy or ethical restrictions.

## Supplementary Materials

The following supporting information can be downloaded at: https://www.mdpi.com/article/10.3390/nu18101484/s1, Table S1: Composition of the VLCKD; Table S2: Clinical characteristics at enrolment. Table S3: Adjusted coefficients (B) with 95% confidence intervals (CIs) and two-sided *p*-values from linear mixed-effects models evaluating longitudinal changes in anthropometric, hemodynamic, and biochemical outcomes according to dietary group (VLCKD vs. LCD). Figure S1: Timeline of assessments; Figure S2: Temporal changes in body mass index according to dietary intervention (VLCKD vs. LCD); Figure S3: Temporal changes in percentage weight loss according to dietary intervention (VLCKD vs. LCD); Figure S4: Temporal changes in aspartate aminotransferase (AST) according to dietary intervention (VLCKD vs. LCD); Figure S5: Temporal changes in alanine aminotransferase (ALT) according to dietary intervention (VLCKD vs. LCD).

## Abbreviations

The following abbreviations are used in this manuscript:

%PWL	Percentage of Pre-Bariatric Weight Loss
ALT	Alanine Aminotransferase
AST	Aspartate Aminotransferase
GGT	Gamma-Glutamyl Transferase
hs-CRP	High-Sensitivity C-Reactive Protein
LCD	Low-Calorie Diet
mo	Months
OSA	Obstructive Sleep Apnea
T2DM	Type 2 Diabetes Mellitus
VLCKD	Very-Low-Calorie Ketogenic Diet
