# Supplementary material for: Preoperative Very-Low-Calorie Ketogenic Diet Versus Low-Calorie Diet in Bariatric Surgery: A Prospective Comparative Study
Source: Nutrients. 2026 May 7;18(10):1484. doi: 10.3390/nu18101484 (PMC13209499; doi:10.3390/nu18101484)
Supplement: Supplementary file 1 [file nutrients-18-01484-s001.zip › Supplementary Figures S3.pdf]

Supplementary figure S3. Temporal changes in percentage weight loss according to dietary intervention (VLCKD vs LCD).

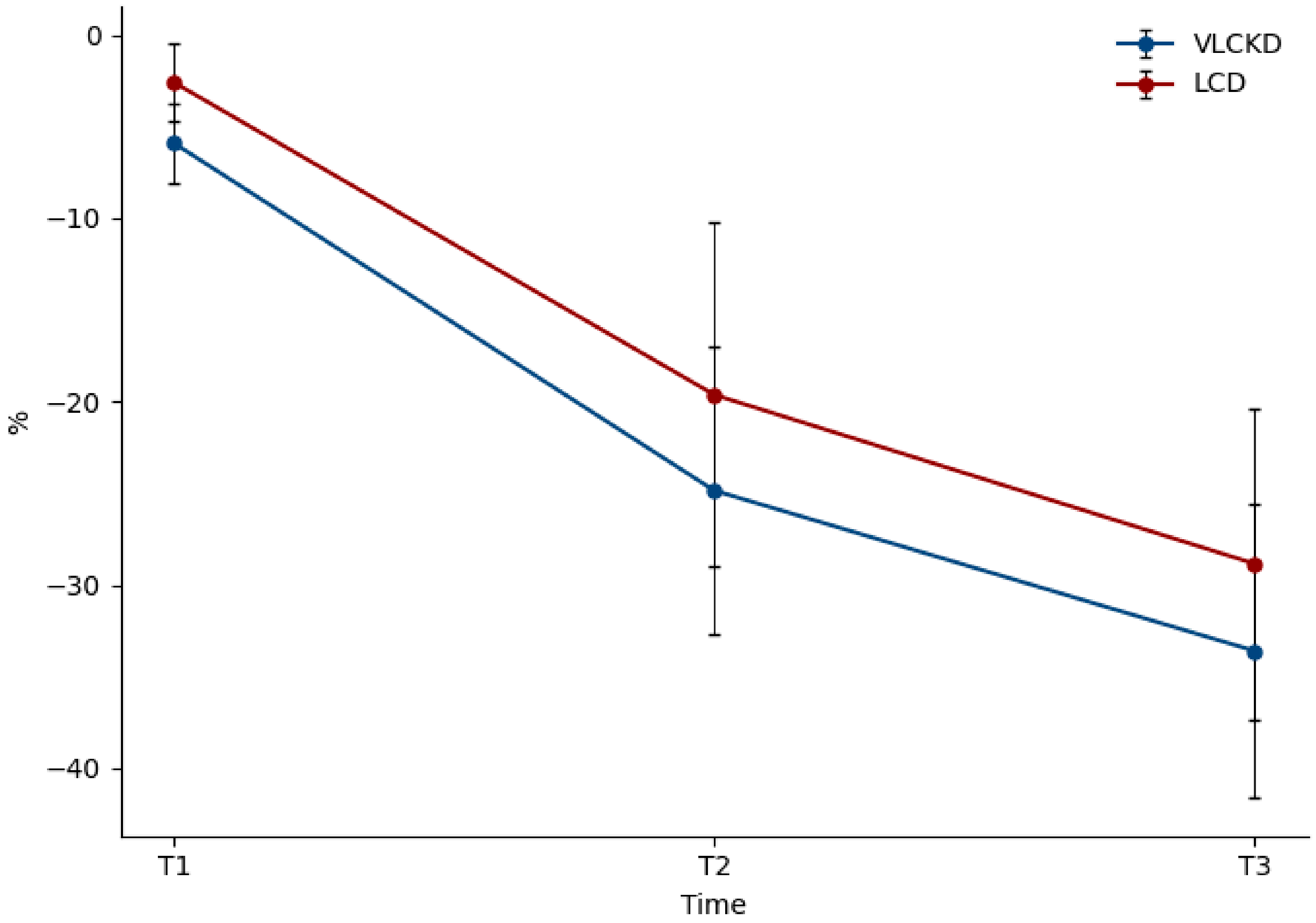

Line plots showing mean ± standard deviation (SD) values for percentage weight loss from baseline over time. VLCKD (very low-calorie ketogenic diet) is shown in blue, while LCD (low-calorie diet) is shown in red. Error bars represent standard deviations. Time points: T1 = 4 weeks, T2 = 6 months after surgery, and T3 = 12 months after surgery.
