# Supplementary material for: Preoperative Very-Low-Calorie Ketogenic Diet Versus Low-Calorie Diet in Bariatric Surgery: A Prospective Comparative Study
Source: Nutrients. 2026 May 7;18(10):1484. doi: 10.3390/nu18101484 (PMC13209499; doi:10.3390/nu18101484)
Supplement: Supplementary file 1 [file nutrients-18-01484-s001.zip › Supplementary Table S1.pdf]

Supplementary Table S1. Composition of the VLCKD

Breakfast
Coffee or tea (no sugar added)<br>AND<br>milk-cream hot drink or biscuit (one pocket: 48 g) or toasted bread (1 portion: 40 g)
Snack (mid-morning and mid-afternoon)
Chips (one portion: 30 g) or bar or crackers (1 pocket: 30g) or veggie mix snack (1 pocket: 35 g)
Lunch and Dinner
Omelet (1 portion: 182 g) or soup (1 bag: 162 g) or pasta (1 portion: 150 g) or sandwich (1 portion: 50 g) or bread (1 portion: 50 g) or tomato cracker (1 pocket: 30 g) or focaccia with olives (1 portion: 60 g)<br>PLUS<br>Vegetables 150 g (vegetables allowed: salad, radicchio, spinach, chard, chicory, fennel, radish, celery, cucumbers, courgettes, cauliflowers, artichokes, chicory, turnip greens, courgettes flowers, lettuce, arugula)
Dressing allowed
Extra-virgin olive oil 20 g
Beverages
Water or drink without sugar 1500-2000 mL
Daily macronutrient intake
Energy 750 kcal, proteins 80 g, carbohydrates 40 g, lipids 30 g, fibers 28 g
Composition of the products per portion
Energy 150-200 kcal, proteins 14-18 g (mainly soy, pea, whey proteins), carbohydrates 4 g, lipids 3-9 g, fiber 5-7g
