# Supplementary material for: Preoperative Very-Low-Calorie Ketogenic Diet Versus Low-Calorie Diet in Bariatric Surgery: A Prospective Comparative Study
Source: Nutrients. 2026 May 7;18(10):1484. doi: 10.3390/nu18101484 (PMC13209499; doi:10.3390/nu18101484)
Supplement: Supplementary file 1 [file nutrients-18-01484-s001.zip › Supplementary Table S2.pdf]

Supplementary Table S2. Clinical characteristics at enrolment

	Total	VLCKD	LCD	P
Number	80	40	40	
Males/females, n	10/70	5/35	5/35	-
Age, years	43.9±10.7	43.8±10.9	43.9±10.6	0.967
Active smoking, n (%)	15 (18.8)	8 (20.0)	7 (17.5)	0.775
T2DM, n (%)	14 (17.5)	7 (17.5)	7 (17.5)	-
Treatment for T2DM, n (%)	11 (13.8)	6 (15.0)	5 (12.5)	0.745
Arterial hypertension, n (%)	28 (35.0)	14 (35.0)	14 (35.0)	-
Treatment for hypertension (%)	17 (21.3)	9 (22.5)	8 (20.0)	0.785
Liver steatosis, n (%)	69 (86.3)	35 (87.5)	34 (85.0)	0.745
OSA, n (%)	17 (21.3)	9 (22.5)	8 (20.0)	0.728
Systolic blood pressure (mmHg)	131.2±13.2	131.3±11.4	131.1±15.0	0.967
Diastolic blood pressure (mmHg)	82.1±8.5	82.0±8.9	82.1±8.2	0.948
Weight, kg	121.9±20.7	122.7±22.2	121.0±19.2	0.710
Height, cm	162.2±7.2	162.4±8.7	162.1±5.6	0.866
BMI, kg/m^2	46.3±7.2	46.4±6.8	46.2±7.8	0.872
Bioelectrical Impedance				
Intracellular water (L)	21.5±3.2	21.7±3.5	21.3±2.8	0.639
Extracellular water (L)	24.7±5.2	24.9±5.3	24.6±5.2	0.794
Fat-free mass (kg)	62.9±10.9	63.4±11.7	62.3±11.3	0.644
Fat mass (kg)	60.0±15.3	59.0±14.9	60.9±15.9	0.586
Waist circumference, cm	125.3±12.8	126.8±11.9	123.8±13.7	0.303
Neck circumference, cm	39.5±4.1	39.8±4.0	39.2±4.2	0.526
Fasting glucose (mg/dL)	95.2±23.9	97.9±31.1	92.6±13.4	0.327
Glycated hemoglobin, mmol/mol	38.8±7.0	38.9±7.5	38.6±6.5	0.824
Triglycerides (mg/dL)	123.2±66.3	121.8±61.2	124.5±71.7	0.858
Creatinine, mg/dL	0.76±0.18	0.75±0.13	0.78±0.23	0.536
AST, U/L	22.0±8.7	22.2±8.8	21.8±8.7	0.818
ALT, U/L	27.6±14.6	27.3±12.1	27.8±16.9	0.867
GGT, U/L	32.7±20.7	34.8±21.5	30.7±20.0	0.374
Hemoglobin, g/dL	13.5±1.2	13.6±1.2	13.5±1.2	0.638
Iron, µg/dL	71.8±30.0	71.3±21.9	72.2±36.6	0.891
Transferrin, mg/dL	296.2±45.8	292.3±42.4	300.2±49.1	0.448
Ferritin, ng/mL	65.0 (25.5; 116.0)	62.0 (23.0; 121.5)	69.0 (28.0; 114.0)	0.627 *
Folic acid, ng/mL	5.1±2.7	4.9±2.0	5.3±3.3	0.569
Vitamin B12, ng/L	397.1±158.6	397.3±159.5	397.0±159.7	0.994
White blood cells, ()	7520.6±2229.7	7463.0±2128.1	7578.3±2352.6	0.819
Hs-CRP, mg/L	8.70 (4.20; 15.3)	8.70 (4.25; 14.6)	8.65 (3.70; 16.0)	0.958 *

Mean±SD; median (25th; 75th quartile); *p-value by Mann-Whitney U test

aspartate aminotransferase (AST), alanine aminotransferase (ALT), -glutamyl transferase (GGT), high-sensitivity C-reactive protein (hs-CRP), low-calorie diet (LCD), obstructive sleep apnea (OSA), type 2 diabetes mellitus (T2DM), very low-calorie ketogenic diet (VLCKD)
