# Supplementary material for: Preoperative Very-Low-Calorie Ketogenic Diet Versus Low-Calorie Diet in Bariatric Surgery: A Prospective Comparative Study
Source: Nutrients. 2026 May 7;18(10):1484. doi: 10.3390/nu18101484 (PMC13209499; doi:10.3390/nu18101484)
Supplement: Supplementary file 1 [file nutrients-18-01484-s001.zip › Supplementary Table S3.pdf]

Supplementary Table S3. Adjusted coefficients (B) with 95% confidence intervals (CI) and two-sided p-values from linear mixed-effects models evaluating longitudinal changes in anthropometric, hemodynamic, and biochemical outcomes according to dietary group (VLCKD vs LCD).

**VLCKD vs LCD**		**BMI**		**Weight**		**Neck circumference**		**Systolic blood pressure**		**Diastolic blood pressure**		**Hs-CRP**	
		B (95% CI)	p-value	B (95% CI)	p-value	B (95% CI)	p-value	B (95% CI)	p-value	B (95% CI)	p-value	B (95% CI)	p-value
Baseline difference		0.9 (-0.5; 2.4)	0.193	2.3 (-3.2; 7.8)	0.405	1.1 (-0.2; 2.4)	0.091	0.5 (-6.4; 7.3)	0.889	-0.6 (-4.6; 3.5)	0.789	0.4 (-2.9; 3.7)	0.801
Time effect	1 mo	-1.1 (-2.3;0.1)	0.062	-3.1 (-6.5;0.4)	0.080	-1.0 (-1.8;-0.2)	0.016	-0.6 (-8.6;7.4)	0.883	-0.2 (-5.5;5.0)	0.925	0.4 (-4.4;5.3)	0.864
	6 mo	-8.7 (-9.8;-7.5)	0.000	-22.9 (-26.3;-19.5)	0.000	-3.6 (-4.4;-2.8)	0.000	-7.6 (-13.2;-2.1)	0.008	-4.0 (-7.6;-0.4)	0.028	-6.3 (-9.5;-3.2)	0.000
	12 mo	-12.6 (-13.8;-11.3)	0.000	-33.9 (-37.7;-30.1)	0.000	-3.9 (-4.9;-3.0)	0.000	-14.1 (-20.4;-7.7)	0.000	-7.0 (-10.7;-3.3)	0.000	-7.4 (-10.5;-4.3)	0.000
VLCKD and time interaction	1 mo	-1.5 (-3.2;0.2)	0.074	-4.2 (-9.0;0.7)	0.093	-0.9 (-2.0;0.3)	0.127	0.3 (-12.5;13.1)	0.961	-0.2 (-8.1;7.6)	0.956	-0.7 (-6.4;5.0)	0.814
	6 mo	-3.0 (-4.7;-1.3)	0.000	-8.1 (-13.0;-3.3)	0.001	-1.3 (-2.4;-0.2)	0.024	-2.8 (-10.6;5.0)	0.480	-0.4 (-5.5;4.7)	0.872	-1.5 (-5.9;2.8)	0.489
	12 mo	-3.4 (-5.2;-1.5)	0.000	-8.8 (-14.0;-3.6)	0.001	-1.6 (-2.9;-0.3)	0.019	-1.0 (-9.8;7.8)	0.817	0.0 (-5.4;5.5)	0.995	-0.9 (-5.2;3.4)	0.674
Covariates	Baseline BMI	0.9 (0.8; 0.9)	0.000	2.0 (1.7; 2.3)	0.000	0.2 (0.1; 0.3)	0.000	0.3 (0.0; 0.6)	0.028	0.1 (-0.1; 0.3)	0.321	0.3 (0.1; 0.4)	0.000
	Age	0.0 (-0.0; 0.1)	0.059	-0.1 (-0.3; 0.1)	0.530	0.0 (-0.0; 0.0)	0.958	0.2 (-0.0; 0.4)	0.065	0.1 (-0.0; 0.2)	0.098	-0.1 (-0.2; 0.0)	0.060
	Male Sex	1.1 (-0.2; 2.3)	0.098	13.3 (7.0; 19.7)	0.000	5.9 (4.5; 7.4)	0.000	3.0 (-2.9; 8.8)	0.314	3.0 (-0.8; 6.8)	0.121	-0.8 (-3.8; 2.2)	0.591
	%PWL	0.2 (0.0; 0.4)	0.018	0.2 (-0.5; 0.9)	0.515	0.0 (-0.1; 0.2)	0.611	0.4 (-0.6; 1.4)	0.396	0.1 (-0.4; 0.6)	0.703	0.5 (0.1; 0.9)	0.018

**VLCKD vs LCD**		**Glucose**		**Triglycerides**		**AST**		**ALT**		**GGT**		**Creatinine**	
		B (95% CI)	p-value	B (95% CI)	p-value	B (95% CI)	p-value	B (95% CI)	p-value	B (95% CI)	p-value	B (95% CI)	p-value
Baseline difference		7.4 (-2.3; 17.1)	0.136	-13.5 (-38.5; 11.5)	0.288	0.0 (-5.5; 5.6)	0.991	-0.8 (-8.8; 7.2)	0.842	2.4 (-6.0; 10.8)	0.575	-0.1 (-0.2; -0.0)	0.010
Time effect	1 mo	-1.7 (-14.2;10.7)	0.780	1.5 (-36.4;39.4)	0.935	-0.1 (-9.4;9.2)	0.980	-0.7 (-13.1;11.6)	0.903	1.0 (-8.9;10.9)	0.843	0.0 (-0.1;0.1)	0.932
	6 mo	-1.6 (-10.4;7.1)	0.712	-17.8 (-39.6;4.0)	0.109	3.9 (-1.2;8.9)	0.133	3.0 (-4.4;10.4)	0.425	-9.0 (-16.1;-1.9)	0.013	-0.0 (-0.1;0.0)	0.552
	12 mo	-5.5 (-14.5;3.4)	0.225	-30.2 (-51.9;-8.6)	0.006	1.4 (-3.8;6.6)	0.588	-5.2 (-12.8;2.4)	0.177	-10.1 (-17.5;-2.7)	0.008	-0.0 (-0.1;0.1)	0.946
VLCKD and time interaction	1 mo	-0.8 (-16.8;15.1)	0.919	-8.0 (-54.7;38.8)	0.733	-1.2 (-11.0;8.5)	0.804	-1.4 (-16.7;13.9)	0.856	-2.5 (-15.9;10.9)	0.708	-0.0 (-0.2;0.1)	0.664
	6 mo	-15.9 (-28.5;-3.4)	0.013	-10.9 (-41.8;19.9)	0.487	-7.2 (-14.3;-0.1)	0.047	-9.8 (-20.1;0.6)	0.064	-4.7 (-15.0;5.5)	0.366	-0.0 (-0.1;0.1)	0.979
	12 mo	-12.5 (-25.1;0.2)	0.054	-8.7 (-39.1;21.7)	0.574	-5.8 (-13.2;1.6)	0.126	-5.3 (-16.1;5.4)	0.330	-5.2 (-15.7;5.3)	0.331	-0.0 (-0.1;0.1)	0.611
Covariates	Baseline BMI	-15.9 (-28.5; -3.4)	0.013	-10.9 (-41.8; 19.9)	0.487	-7.2 (-14.3; -0.1)	0.047	-9.8 (-20.1; 0.6)	0.064	-4.7 (-15.0; 5.5)	0.366	-0.0 (-0.1; 0.1)	0.979
	Age	-12.5 (-25.1; 0.2)	0.054	-8.7 (-39.1; 21.7)	0.574	-5.8 (-13.2; 1.6)	0.126	-5.3 (-16.1; 5.4)	0.330	-5.2 (-15.7; 5.3)	0.331	-0.0 (-0.1; 0.1)	0.611
	Male Sex	0.8 (0.3; 1.3)	0.001	0.4 (-0.7; 1.6)	0.466	0.2 (-0.0; 0.5)	0.083	0.2 (-0.2; 0.6)	0.253	0.1 (-0.3; 0.6)	0.508	0.0 (0.0; 0.0)	0.000
	%PWL	0.1 (-0.2; 0.4)	0.686	-0.2 (-0.9; 0.4)	0.477	-0.1 (-0.2; 0.1)	0.447	-0.0 (-0.3; 0.2)	0.762	-0.1 (-0.4; 0.2)	0.423	0.0 (0.0; 0.0)	0.018

Fixed effects included diet group (VLCKD = 1, LCD = 0), time (baseline [reference], 1 month, 6 months, and 12 months), and the Diet × Time interaction. Models were adjusted for baseline BMI, age, sex, and percentage of pre-bariatric weight loss (%PWL). A random intercept for participant was included to account for within-subject correlation.

"Baseline difference" represents the adjusted difference between VLCKD and LCD at baseline. "Time effect" represents the mean change from baseline over time in the reference group (LCD). "VLCKD × Time interaction" represents the additional change in the VLCKD group beyond the time effect observed in the LCD group (i.e. difference-in-differences).

Accordingly, the between-group difference at each time point is calculated as: baseline difference + VLCKD × Time interaction; the within-VLCKD change over time is calculated as: time effect + VLCKD × Time interaction.

Models were estimated using maximum likelihood under the missing-at-random assumption within an intention-to-treat framework.

Units: BMI (kg/m^2); body weight (kg); neck circumference (cm); systolic and diastolic blood pressure (mmHg); hs-CRP (mg/L); glucose, triglycerides, and creatinine (mg/dL); AST, ALT, and GGT (U/L).

Abbreviations: B, beta; 95%CI, 95% confidence intervals; VLCKD, very low-calorie ketogenic diet; LCD, low-calorie diet; %PWL, percentage of pre-bariatric weight loss; hs-CRP, high-sensitivity C-reactive protein; mo, months.
