# Supplementary figures and images for: Preoperative Very-Low-Calorie Ketogenic Diet Versus Low-Calorie Diet in Bariatric Surgery: A Prospective Comparative Study
Source: Nutrients. 2026 May 7;18(10):1484. doi: 10.3390/nu18101484 (PMC13209499; doi:10.3390/nu18101484)

**Supplementary Figure S1. Timeline of assessments**

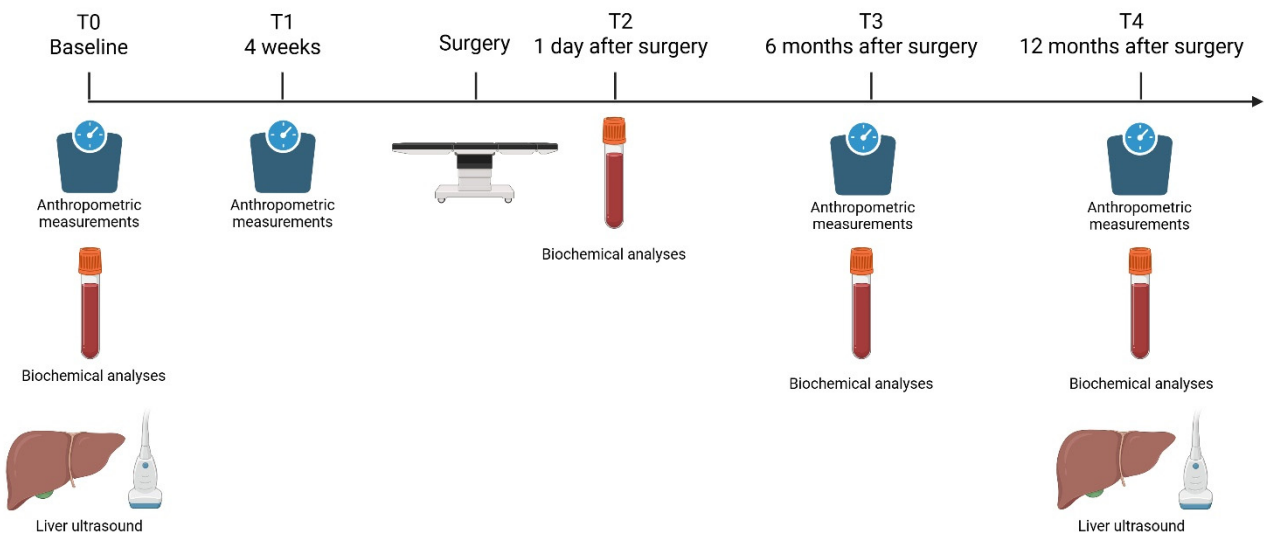

Supplement: Supplementary file 1 [file nutrients-18-01484-s001.zip › Supplementary Figure S1.pdf]
